# Oncogenic Pathways in Neurodegenerative Diseases

**DOI:** 10.3390/ijms23063223

**Published:** 2022-03-17

**Authors:** Luis Varela, Maria E. R. Garcia-Rendueles

**Affiliations:** 1 Yale Center for Molecular and Systems Metabolism, Department of Comparative Medicine, School of Medicine, Yale University, 310 Cedar St. BML 330, New Haven, CT 06520, USA; 2 Precision Nutrition and Cancer Program, IMDEA Food Institute, Campus Excelencia Internacional UAM+CSIC, 28049 Madrid, Spain

**Keywords:** cancer, neurodegenerative disease, Alzheimer, Parkinson, Huntington, hippo, notch, NRF2, PI3K, p53, cell cycle, WNT, TGFβ, MYC, MAPK

## Abstract

Cancer and neurodegenerative diseases are two of the leading causes of premature death in modern societies. Their incidence continues to increase, and in the near future, it is believed that cancer will kill more than 20 million people per year, and neurodegenerative diseases, due to the aging of the world population, will double their prevalence. The onset and the progression of both diseases are defined by dysregulation of the same molecular signaling pathways. However, whereas in cancer, these alterations lead to cell survival and proliferation, neurodegenerative diseases trigger cell death and apoptosis. The study of the mechanisms underlying these opposite final responses to the same molecular trigger is key to providing a better understanding of the diseases and finding more accurate treatments. Here, we review the ten most common signaling pathways altered in cancer and analyze them in the context of different neurodegenerative diseases such as Alzheimer’s (AD), Parkinson’s (PD), and Huntington’s (HD) diseases.

## 1. Introduction

Cancer and neurogenerative diseases are two of the leading causes of death in modern societies, and despite all the efforts made in understanding their onset and development, their prevalence continues to increase dramatically. Cancer, together with cardiovascular disease, is known to be the main cause of premature death [1]. In the last years, its incidence has risen rapidly. It reached an estimated 20 million new cases worldwide within 2020, and it is believed that in the next two decades, the incidence of cancer will increase by 50%. Cancer was also the cause of 10 million deaths last year [2]. In line with this, neurodegenerative disorders are a group of age-related diseases [3] that affect millions of people worldwide. They have become an important public health burden, with increasing incidence and mortality and an associated rise in healthcare costs [4,5]. The fact that aging is the major risk for neurodegeneration, and together with the expectations that the aged population will exceed the number of young individuals in the next decades, makes neurodegenerative diseases one of the most important threats to the well-being of individuals and society.

Although the causes and consequences of the different neurodegenerative diseases are various, their common clinical features are marked by a progressive loss of cognitive function, defective motor coordination, and increased pain triggered by, in all cases, a loss of specific neuronal populations (Table 1) [6]. Alzheimer’s (AD) and Parkinson’s (PD) diseases are the two most prevalent neurodegenerative disorders, and they are characterized by the aberrant accumulation of aggregates, Amyloid β (Aβ) in senile plaques and Tau in neurofibrillary tangles in AD, and α-synuclein in Lewis bodies in PD [7,8]. Despite the hallmarks of AD and PD being identified, the mechanisms underlying their development remain far from completely understood. Altered oxidative stress, cell cycle activation, and inflammation, among others, are the stimuli that trigger neurodegeneration [9,10]. Although rare, accounting for 5% of all the cases, mutations in specific genes are the cause of familial AD and PD. *APP*, *APOE*, *PARKIN*, and *PINK*1 have been identified as causal genes of AD and PD [11,12]. On the contrary, Huntington’s disease (HD) is a hereditary disease caused by the mutation of *HTT* [13].

In cancer, cells acquired the ability to divide and growth uncontrollably [33,34,35]. Unlike normal cells, cancer cells do not respond to the controlling signals mainly due to molecular alterations in specific genes associated with signalling pathways. The complexity is since, all these signalling routes are connected forming an intricated signalling network, thus oncogenic mutations can affect proteins implicated in several signalling pathways, such as Notch-Wnt-TGFb-Hippo pathways. Moreover, there is considerable variation in the genes and pathways altered across different tumor types and individual tumor samples. 

Although the mechanisms underlying cancer and neurodegenerative disorders are different, the onset and progression of both diseases share the same molecular signaling pathways. In this review, we provide a summary of the molecular alterations implicated in neurodegenerative diseases, based on the ten canonical signal pathways most altered in cancer [36]. The objective is to understand the role of critical cancer pathways in neurodegenerative diseases.

## 2. Oncogenic Signaling Pathways

### 2.1. Hippo Pathway

First discovered as a regulator of organ size, Hippo signalling is involved in many different processes, such as mechanotransduction, homeostasis, cellular differentiation, and tissue regeneration, among others [37,38,39]. Briefly, the canonical Hippo pathway induces the activation of MST1/2 (mammalian sterile 20-like kinases 1 and 2) which, through the phosphorylation of LATS1/2 (large tumor suppressors 1 and 2), phosphorylates YAP/TAZ. Phosphorylated YAP (Yes-associated protein) is retained in the cytoplasm and marked for proteasomal degradation. Non-phosphorylated YAP is translocated to the nucleus, where it complexes with different transcription factors to initiate the transcription of genes involved in cell proliferation and survival [39,40]. YAP overexpression and nuclear localization have been described in different cancers due to the inactivation of the Hippo pathway or the constitutive activation of YAP. This aberrant location of YAP drives the transcription of genes involved in metastasis, pro-tumoral microenvironment, or anti-apoptosis [41,42]. Mutations in Hippo components are rare in cancer [43,44] ], NF2 being the most mutated one. Germline heterozygotic mutations in NF2 cause neurofibromatosis type 2 that predisposes to tumors of the nervous system such as schwannomas, meningiomas, and ependymomas [45,46]. YAP nuclear localization and activation are the principal cause of tumorigenesis and drug resistance [47,48].

The Hippo pathway has been well-studied in the developing brain (reviewed in [49,50]), but its relevance in the adult brain has emerged recently. Hippo pathway components have been suggested as early markers of degenerative diseases within the developing brain [51]. Different integrated analysis studies of postmortem brains revealed the downregulation in Hippo pathway-related genes in various brain areas of AD patients [52,53]. In line with these findings, in AD mouse models, YAP mRNA expression is downregulated in the earlier stages of the disease. The subcellular location of YAP was found altered in postmortem brains of MCI and AD patients [54]. The appearance of Aβ aggregates sequestrates YAP in the cytoplasm of cortical neurons, reducing the accumulation of YAP in the nucleus of these neurons. The mouse models of AD 5xFAD and amyloid precursor protein (APP) knock-in [54] present YAP cytoplasmatic location even before the onset of cognitive impairments. Interestingly, overexpression of YAP, by administration of AAV-YAPdeltaC61 into the cerebrospinal fluid (CSF) space, increased the levels of nuclear YAP, decreased extracellular Aβ plaques, and restored different behavioral parameters of 5xFAD mice to levels similar to control mice [54]. 

Changes in YAP location are not exclusive of AD; it also has been found in postmortem brains of Huntington’s disease patients as reported [55,56]. In cortical neurons of HD patients, YAP is localized mainly off the nucleus. Mouse models revealed increased levels of total YAP and phosphorylated YAP in the striatum and cortex [55]. Interestingly, cytoplastic YAP localization in the neurons of AD and HD patients has been linked to a new mechanism of necrosis, TRIAD (TEAD-YAP dependent necrosis) [56]. TRIAD, characterized by ER enlargement, has been found in different mouse models of neurodegenerative diseases. Sequestration of YAP in the cytoplasm seems to drive the appearance of ER ballooning. Abnormal morphology of the ER is reversed by specific overexpression of YAP [54].

Although YAP has lately gained relevance as the main effector of the Hippo signaling in the onset of neurodegenerative disorders, other components of the pathway, such as MST1 and LATS1/2, have been also identified and linked to the progression of different CNS diseases [57,58]. For example, higher levels of phospho-MST1 were reported in the motor neurons of the spinal cord in both ALS patients and animal models [58]. In PD, MST1 is involved in dopaminergic neuronal loss. Activated MST1 phosphorylates UNC5B, a pro-apoptotic netrin family receptor, causes motor dysfunctions and reduced dopaminergic cell counts in the substantia nigra (SN) [57]. Similar to YAP, MST1 was found overexpressed in the postmortem brains of HD patients [55]. 

### 2.2. Notch Pathway

Notch is a conserved pathway responsible for a wide range of physiological roles, including self-renewal, differentiation, angiogenesis, and proliferation [59,60]. Notch activity is reported to have complex and context-dependent effects [61]. The canonical Notch pathway includes the activation of the Notch family receptors (Notch 1, 2, 3, and 4) by the binding of DSL ligands that induce the cleavage of Notch in NECD (Notch extracellular domain) and NICD (Notch intracellular domain) [62,63]. NICD is translocated into the nucleus where it forms a complex with Mastermind-like (MAML) and other co-activators to stimulate the transcription of Notch target genes. Mutations in Notch receptors have been found in T-cell acute lymphoblastic leukemia, breast cancer, and adenoid cystic carcinoma [64,65,66,67]. 

The Notch signaling pathway regulates neurogenesis, neural maturation, and synaptic plasticity in the brain [68,69,70]. Moreover, in the adult brain, Notch has been shown to play an important role in the formation of Aβ plaques [70]. Notch colocalizes with presenilins (PSs), the catalytic component of γ-secretase that cleaves APP and induces the aggregation of Aβ [71,72]. Different studies have detected abnormal expression of Notch in the postmortem brains of AD patients [73,74]. Findings from the 1990s pointed at neurons as the key players of this aberrant expression of Notch [73]. The first studies identified aberrant expression of Notch in the hippocampus of AD patients and pointed at neurons from different areas as the main sites of expression of Notch. Recently, a series of works have focused in more detail on the location of Notch in AD patients. In a very elegant study, Brai et al. [75] described that the increased expression of Notch in the postmortem brains of AD patients was due to their aggregation in plaque-like structures. Neurons from different cortical and hippocampal areas presented lesser expression of Notch due to the specific decrease of the extracellular domain of Notch levels. The authors also showed that NECD accumulated in Aβ plaques in brains of AD patients. Interestingly, all the Notch- and Aβ-positive plaques were invaded by microglia and astrocytes, suggesting a potential involvement of these cells in pro-inflammatory response to Notch delocalization [75]. Moreover, AD patients presented less Notch expression in the CSF than healthy patients [70]. Notch accumulation in plaque-like structure in the parenchyma reduced the filtration to the CSF.

Notch ligands were also involved in the onset and development of different neurodegenerative diseases. Among others, alterations in Jagged1 were observed in AD patients [76]. Like the specific reduction of Notch in hippocampal and cortical neurons, AD patients presented fewer Jagged1 expression levels. The generation of a mouse model with a deleted expression of Jagged in neurons showed a potential role of Notch ligand in memory loss. Specifically, this animal model has a reduced expression of Notch in hippocampal neurons and presented an impaired spatial memory similar to the observed in AD patients [29].

Although to a lesser extent, Notch signaling is also related to other neurodegenerative diseases, such as PD and Down syndrome [77,78].

### 2.3. Nrf2 Pathway

Nrf2 (Nuclear factor-erythroid factor 2-related factor 2) holds key physiological functions in homeostasis maintenance and cell proliferation. It is a master regulator of redox balance and antioxidant-related activity [79]. Recently, its role in metabolic reprogramming was described [80]. Under basal conditions, Nrf2 is sequestered with Keap1 (Kelch-like ECH-associated protein 1) and leads to CUL3-mediated ubiquitination followed by proteasome degradation [81]. Under upstream signals, such as oxidative stress, Nrf2 dissociates from Keap1, translocates to the nucleus, complexes with ARE and other transcription factors, and induces the transcription of detoxification, antioxidant, metabolism, or proliferative genes [82,83]. Constitutive Nrf2 nuclear localization and hyperactivation correlates with cancer progression and chemoresistance, in glioblastoma, lung, hepatocellular carcinoma, cervical, and pancreatic cancer [84]. Nrf2 activation inhibits apoptosis and increases proliferation and invasion [85,86].

Nrf2 has been widely studied within the CNS [87,88,89]. Despite the different neurodegenerative diseases having diverse causes and consequences, oxidative stress is a common pathogenic mechanism in such disorders. In this sense, Nrf2 emerges as a crucial factor in oxidative damage response found in the early stages of AD and PD [90,91]. Nrf2 antioxidant effects have been proposed as a therapeutical target for the treatment of neurological disorders. Nrf2’s role in AD and PD was first reported, in the study of postmortem brains of AD and PD patients [92]. There are differences in the Nrf2 subcellular location between AD and PD brains. Whereas in hippocampal neurons from AD patients the Nrf2 staining was mainly cytoplasmatic, in the SN of PD patients, dopaminergic cells exhibited a strong nuclear location of Nrf2 [92]. Although the reason for these differences in the subcellular location of Nrf2 remains unknown, one possible explanation given by the authors of the study might settle on the dynamics and the timing of the disease. In situations of enhanced oxidative stress, Nrf2 is translocated to the nucleus, where it triggers the transcription of genes involved in the antioxidant response in neurons [92]. The fact that in AD Nrf2 is found mainly in the cytoplasm is a sign of disrupted acclimation of neurons to the oxidative conditions. On the other hand, it is known that PD produces dopaminergic cell loss. The nuclear location of Nrf2 in the SN of PD patients could probably be seen in the neurons that still maintain proper functions, while those dead dopaminergic cells have no Nrf2 staining. 

There are numerous efforts to determine the relevance of Nrf2 in the development of neurodegenerative disorders using transgenic mouse models. AD mouse models (APP/PS1 mouse) showed defective expression of Nrf2 and its downstream targets in the hippocampus and cortex, coinciding with the increase of Aβ aggregates [93,94]. Interestingly, lentivirus-meditated overexpression of Nrf2 in the hippocampus of APP/PS1 mouse improves the learning deficits while reducing the levels of soluble Aβ of this mouse strain [95]. Loss of Nrf2 also exacerbates the effects of other AD mouse models in Aβ deposition and spatial learning and memory [93]. 

Nrf2 involvement in the progression of neurodegenerative disorders has also been linked to neuroinflammation and autophagy. p62, an autophagy marker, is closely related to the Nrf2 signaling pathway [88,96]. Loss of this function impairs the clearance of Aβ aggregates. p62 accumulates in the cytoplasm in absence of autophagy and interacts with the complex t Nrf2-Keap1 [89,97]. p62 releases Nrf2 from Keap1 and is translocated to the nucleus to induce the transcription of antioxidant enzymes and autophagy-related genes. Interaction between p62 and Nrf2 is a positive feedback loop, where defective autophagy [97] activates the oxidative stress response and autophagy itself. The imbalance of this mechanism has been shown to be relevant in the progression of neurological disorders.

### 2.4. WNT/β-Catenin Pathway

The Wnt/β-catenin pathway is a highly conserved pathway that regulates key cellular functions including gene stability, differentiation, proliferation, apoptosis, stem cell renewal, and migration [98,99,100]. The canonical pathway consists of WNT proteins binding to frizzled receptors and LRP co-receptors, suppressing the activity of the “β-catenin destruction complex” and free β-catenin. This complex is composed of APC (adenomatous polyposis coli), Axin, CK1α (casein kinase 1α), and GSK-3β (glycogen synthase kinase-3β). β-catenin translocates to the nucleus where it associates with Tcf/Lef inducing the transcription of proliferation genes, such as c-Myc, cyclin D1, or c-JUN [101,102,103]. In the absence of WNT ligand, “β-catenin destruction complex” is active, recruit -TrCP E3 linker (β-transducin repeat-containing protein, an E3 ubiquitin ligase), and subsequently degrade β-catenin via the proteasome. In the cytoplasm, β-catenin could form complex with adherent junctions and promotes cell-to-cell adhesion. This pathway has been found altered in cancer and is involved in initiation, progression, and metastasis, involving CSC (cancer stem cell) activation. APC mutations have been found in 90% of colorectal, 70% of gastric cancers, 50% of liver cancers, and 5% of colorectal [100]. Melanoma, prostate, thyroid, and ovary showed mutations in β-catenin [100]. CTNNB1 mutations are present in 90% of WNT-activated medulloblastomas [104]; mutations in β-catenin, APC and AXIN1 have also frequently been identified in medulloblastoma [105,106,107].

Despite the well-known role of WNT signaling in the developing brain [108,109], where it controls synapse formation or neurogenesis, its function in the mature brain is not fully unraveled. Different studies have shown that several components of the WNT signaling are altered in age-related disorders and have been linked to tau and amyloid pathologies, hallmarks of AD disease [110]. 

Different players of the WNT pathway are altered in the postmortem brains of AD patients [111]. DKK1, an extracellular ligand of WNT receptors and negative regulator of the pathway, is highly expressed in cortical neurons of the diseased brain [112,113]. LPR6, co-receptor of the WNT signaling, is downregulated in the temporal cortex of AD patients. Interestingly, LRP6 downregulation is associated with a lower expression of β-catenin, and hence, to a lower translocation of this protein to the nucleus, where it activates WNT target genes [110].

Different mouse strains of neurogenerative disease also showed altered expression of WNT components [94,110,112]. Mouse models of amyloid deposition and tau pathology showed increased expression of DKK1, accompanied by lower levels of β-catenin. Interestingly, the important role of WNT pathway in the formation of Aβ plaques has been demonstrated in different mouse models. Loss of WNT signaling exacerbates the amyloid deposition in mouse models of amyloid pathology [113]. 

In postmortem brains derived from patients suffering from other neurodegenerative disorders, such as Parkinson’s and Huntington’s diseases, scientists have found dysregulation of the WNT pathway. For example, genes regulated by WNT were found downrated in the SN of PD brains [111,113,114]. The mechanism by which WNT affects dopaminergic cell loss in SN of PD patients is linked to its role in synapse formation and cell regeneration.

### 2.5. TGFβ Pathway

The TGFβ (transforming growth factor β) superfamily of growth factors includes TGFβ, activins, and BMPs. The activated TGFβ ligands interact with type II TGFβ receptors (TβRII), which subsequently recruit and phosphorylate type I TGFβ receptors (TβRI), thereby activating downstream signaling through either the SMAD-dependent canonical pathway or the SMAD-independent non-canonical pathway. The canonical pathway involves R-Smad (Smad2/3) phosphorylation complex with co-Smad (Smad4) to translocate to the nucleus and activate the expression of genes [115,116]. The non-canonical pathway activates other upstream components of different pathways such as tumor necrosis factor (TNF) receptor-associated factor (TRAF) 4 or TRAF6, TGFβ-activated kinase 1 (TAK1), Rho GTPases, mitogen-activated protein kinase (ERK or p38), jun N-terminal kinase (JNK), or nuclear factor-κB (NF-κB) [117,118,119]. 

In normal cells, the TGFβ pathway regulates key physiological functions in homeostasis, development, tissue, and cell growth [120]. In cancer, TGFβ has opposite roles. While in early stages, TGFβ acts as a tumor suppressor by inducing apoptosis and promoting cell-cycle arrest; in advanced-stage cancers, TGFβ acts as a tumor promoter [120,121,122,123]. Cancer cells escape to growth control by mutations or epigenetic modifications in the components of the TGFβ signaling cascade or by becoming resistant to the suppressive effects of TGFβ signaling [120,121,122,123]. TGFβ turns into an oncogenic factor and induces proliferation, epithelial to mesenchymal transition (EMT), evasion of immune surveillance, angiogenesis, drug resistance, and cancer cell stemness. High levels of TGFβ in patients with breast, lung, colorectal, and thyroid cancer have been described, and it is a prediction for poor prognosis [124,125,126,127,128,129]. 

The TGFβ superfamily is involved in neuroinflammation and repair after brain injury [130]. In the CNS, the TGFβ pathway components have low expression. Astrocytes are the principal source of TGFβ, while TGFβ receptors are in the neurons of different restricted areas [131]. It has been shown that TGFβ plays an important role in age-dependent diseases. Aging increases TGFβ expression in the brain [132]. Although many works have studied this pathway in the pathology of different neurodegenerative disorders, its exact role is not completely understood. Some studies showed TGFβ’s beneficial role in the onset of AD, PD, and other diseases, and others have reported its detrimental effects. Similar to cancer, TGFβ seems to play a dual role in neurogenerative disorders depending on the specific context [130]. TGFβ pathway has been shown altered in brain, CSF, and blood of human AD, PD, and HD patients [132,133,134,135,136]. In AD patients, TGFβ is decreased in plasma but increased in CSF [132], TβRII expression is reduced in the brain, the phosphorylation of SMAD2/3 and its subcellular location are also altered in the diseased brain [137]. Incongruity has also been found in HD, where some reports described increased plasmatic levels of TGFβ [138], and others found lower levels of TGFβ in blood [133].

In the AD brain, the TGFβ pathway promotes amyloid deposition and is co-expressed with Tau in neurons and tangles [139]. TβRII silencing in cortical and hippocampal neurons decreased TGFβ activity and triggered neurodegeneration. TβRII deficient mice showed a smaller number of neurons but a higher number of astrocytes [131]. The TGFβ role in the pathogenesis of the disease was corroborated by breeding the mice losing TGFβ signaling to mouse models of AD. Interestingly, the combination of both models induced an enhanced accumulation of Aβ plaques due to increased levels of APP found in the neurons of this model [131]. A pharmacological study showed that central administration of TGFβ reduced plaque formation and rescued the Aβ induced cognitive impairment [140]. Other works, however, have described that overexpression of TGFβ induces the onset of amyloid pathology [141,142].

TGFβ seems to be involved in the onset and development of Tau pathology. TGFβ levels have been correlated with NFTs in AD brain. Moreover, Smad2/3 co-expressed with Tau in both neurons and NFTs [137,139].

TGFβ has different implications in the development of PD. It is increased in the brains of PD patients [143]. Several transgenic mouse models losing TGFβ signaling presented a reduced number of dopaminergic cells within the SN [144], suggesting that impairment of the TGFβ signaling increases the risk of PD. Although opposite effects of TGFβ have been described, a recent study shows that loss of TGFβ signaling in neurons shows age-related memory and cognitive deficit and presents an important sign of degeneration in the SN. Overexpression of TβRI in the SN of wild type-mice protected against MPTP (1-methyl-4-phenyl-1,2,3,6-tetrahydropyridine) -induced neurodegeneration and cognitive loss [144].

Although to a lesser extent, a potential role of TGFβ in the cortical neurons of HD patients has been described [145]. TGFβ signaling was also found altered in Pick’s disease and ALS, involving the subcellular location of Smad2/3 [137].

### 2.6. MYC Pathway

C-Myc belongs to the MYC family that also encompasses N-Myc and L-Myc proteins in mammalian cells. Although highly homologous, they display more tissue-restricted expression [146]. c-Myc heterodimerizes with MAX protein, and the complex binds “E-boxes” with a consensus sequence 5′-CACGTG-3′, enriched in the promoters and enhancers to regulate gene expression [147,148]. c-Myc plays a role as a signal node, so under normal circumstances, its expression is tightly regulated by important pathways, such as Wnt/beta-catenin, Ras/Raf/ERK, and the Ras/PI3K/AKT/GSK-3 pathways. As a transcription factor, c-Myc responds to and integrates these signals into broad changes in gene expression, supporting cell growth, proliferation, apoptosis, energy metabolism with biomass accumulation, and diverse biosynthetic pathways [149,150,151,152,153]. In malignant cells where c-Myc is overexpressed, c-Myc can bind DNA sequences beyond E-boxes [154].

Myc is frequently dysregulated during tumorigenesis and is a central driver in multiple cancers, such as breast cancer [155], liver tumor [156], colorectal carcinoma [157], and prostatic neoplasia [153]. Either high, aberrant, or in combination, Myc expression occurs in >70% of human cancers and is related to poor prognosis and aggressive conditions [158,159,160]. MYC alterations have been found in cancer but were mutually exclusive with PIK3CA, PTEN, APC, or BRAF alterations, suggesting that MYC is a distinct oncogenic driver [161].

Although the characterization of the MYC members has been very extensive in the study of different cancer types, its role in neurodegenerative diseases is still far from being understood. Similar to its functions in cancer progression, MYC has also been tightly linked with cell cycle re-entry in the onset and development of AD and other CNS disorders [162].

Alterations of the different Myc members were found in the brains of AD and HD patients [163,164]. Whereas n-Myc expression is decreased, specifically in AD brains, HD seems to affect only the c-Myc expression in PD brains, there is no difference in the expression pattern of Myc members [164]. A subsequent study analyzes the phosphorylation of c-Myc and its subcellular location in AD. Interestingly, despite no differences being reported in total c-Myc expression in the hippocampus of AD brains, phosphorylation status seems to be altered [164]. Phospho-c-Myc was found in NFT positive neurons and in the vicinity of senile plaques in AD, Pick’s disease, and other neurogenerative disorders [164]. Increased mRNA levels of c-Myc were found in the human brain and a mouse model of AD [165].

Insights into the relevance of c-Myc in the onset of neurodegenerative diseases were first reported by a study that generated a mouse strain expressing c-Myc in neurons [166]. Increased expression of c-Myc triggered neuronal loss in the hippocampus and memory deficits. Moreover, another study showed the role of n-Myc in CNS disorders development. Ablation of NDRG2, n-Myc downstream gene 2, exacerbates the AD-like phenotype in pharmacological and genetic models of AD [167]. NRG2 levels were affected by Aβ in a mouse model of AD [167]. Increasing levels of NRG2 were linked to increased expression of APP and the appearance of Aβ plaques [167].

### 2.7. MAPK Pathway

The mitogen-activated protein kinase (MAPK) is a complex interconnected signaling cascade that converges in the amplification of key molecules that sustain cell proliferation, growth, and survival processes [168]. The MAPK family consists of mainly four signaling families activated by receptor tyrosine kinases (TRKs): MAPK/ERK, the Big MAP kinase-1 (BMK-1), c-Jun N-terminal kinase (JNK), and p38 signaling families. The canonical pathway involves ligand-bound RTKs that activate RAS (GTPases family HRAS, KRAS, and NRAS) with the consequent activation by phosphorylation of RAF family members (ARAF, BRAF, and CRAF), MEK and ERK, the final effector. Nuclear pERK activates the transcription of survival, proliferation, and differentiation genes [169,170].

The MAPK pathway is one of the most altered pathways in cancer. Braf is altered in 60% of thyroid cancer, 54% of melanoma, and 11% colorectal cancers [36,171,172]. Mutations in BRAF^V600E^ are by far the most identified mutation in human tumors. This mutation results in strong BRAF kinase activation (independent of upstream signal) and constitutively hyperactivation of the MAPK pathway. Approximately 19% of patients with cancer harbor RAS mutations, with KRAS responsible for 75% of that number. Furthermore, 65% of pancreatic cancers harbor an RAS mutation, 47% of colorectal, 30% of melanoma and lung cancers, and 12% of thyroid cancer. KRAS is the main isoform mutated in pancreatic and colorectal, NRAS in melanoma and thyroid carcinoma. Moreover, mutations also occur in the genes coding for the tyrosine kinase receptors (EGFR, c-MET, c-KIT) [36,171,172]. EGFR is frequently altered in glioblastoma: 44% presented EGFR amplification, and 23% harbor EGFR mutations [36,171,172]. 

The canonical ERK cascade, the p38 and the JNK pathways play an important role in different neurological disorders. In the brain, the MAPK pathway is involved in neurogenesis, gliogenesis, and synapse transmission, which affects cognition and memory formation. Pharmacological and genetic approaches showed that alteration of the different MAPK pathways leads to changes in behavior in terms of cognition and learning, incipient signs of neurological disorders [173]. 

In human patients with AD, PD, HD, or other neurological disorders, increased levels of activated ERK, p38, and JNK have been found. Higher pMEK and pERK levels were found in AD [174,175,176] and PD [177] brains at different stages. The active form of p38 (phosphorylated) is upregulated in the early stages of AD [178,179]. JNKs were reported to increase not only in different areas of the brains of AD patients but also in their CSF. JNKs play a relevant role in the dopaminergic cell loss characteristic of PD. Furthermore, in several models of AD and PD, the different MAPK signaling pathways were demonstrated to be upregulated [180].

In AD, MAPK pathways are linked to both amyloid and tau pathologies. In fact, in the brains of AD patients, different members of the cascade members were found to co-express in NFTs and senile plaques. Moreover, pharmacological blockade and genetic deletion of pERK, p38 and JNK ameliorate the cognitive impairments in different mouse models of AD [180,181,182,183,184,185]. These restored memory and learning functions are associated with diminished levels of Aβ accumulation. Several reports have linked the expression of APP with the activity of the MAPK pathway. For example, lower amounts of Aβ deposition driven by the loss of p38 in an AD model are associated with a decrease in the β-secretase activity. Another study [185] showed that JNK inhibition also ameliorates working memory, and this is associated with a reduction of plaques in cortical, and hippocampal areas, lower levels of secretase activity, and diminished expression of phosphorylated APP.

ERK, p38, and JNK pathways are involved in the survival of dopaminergic cells in the striatum and the dopaminergic signaling of the brain [186]. It has been shown that pharmacological models of parkinsonism have altered the expression of all the most important MAPK pathways [185,186]. Regarding the genetic ablation of JNK2 protected against the MPTP-induced Parkinson model, this study is the first to show the possible relevance of MAPKs in the development of PD [186]. Moreover, pharmacological blockade of another isoform of JNK (JNK3) mitigates the dopaminergic cell loss induced by MPTP [181]. p38 is involved in the dopaminergic cell loss by responding to dysregulated oxidative stress in these neurons. It was also described that α-synuclein induces the expression of p38, ERK, and JNK in glial cells [187,188]. α-synuclein released by damaged neurons signals to microglia and triggers and pro-inflammatory responses of these glial cells [188].

At the onset and development of HD, MAPK may also play an important role. Like humans, mouse models mimicking the effect of HD [189] presented increased levels of phosphorylated p38 and JNK in the striatum. Different studies in cell cultures suggest that mutant HTT affects MAPK pathways and activates them [190]. 

### 2.8. p53 Pathway

p53 is a potent tumor suppressor, and it is considered the “guardian of the genome” to prevent the accumulation of oncogenic mutations that lead to malignant tumors [191,192]. p53 is the principal responder to various cellular stress signals, such as hypoxia, oxidative stress, oncogenic activation, DNA damage, ribosomal stress, and telomere erosion. The tetrameric transcription factor p53 is activated through multiple phosphorylation events and, depending on the type of stress, this activation results in upregulation or repression of genes involved in cell cycle arrest to restore genetic integrity and DNA repair, apoptosis, senescence, autophagy, or ferroptosis to eliminate unrecoverable cells. p53 also regulates genes involved in anti-angiogenesis, protection against oxidative stress, the regulation of metabolic homeostasis, and stem cell maintenance roles. In unstressed cells, p53 protein levels are regulated via a negative-feedback loop, whereby p53 induces the transcription of its own negative regulator, MDM2, that ubiquitinates p53 and marks it for proteasomal degradation. p53 is inactivated in almost every tumor, through either mutation in the p53 gene or the deregulation of its associated pathways [191,192,193]. The majority of p53 mutations are missense mutations that lead to the synthesis of p53 proteins unable to bind the target gene promoters of wild-type p53 [194]. Mutated p53 can sequester various tumor suppressors, including non-mutated p53 (dominant-negative function) and the family members p63 and p73, inhibiting their pro-apoptotic function [195]. Most p53 missense mutants acquire oncogenic gain-of-function activities that allow them to interact with other transcription factors, including NF-Y, Sp1, ETS1/2, NF-kB, and SMADs [196]. These changes lead to increased genetic instability, cellular proliferation, metastasis, and chemo-/radio-resistance [197]. 

p53 has been widely involved in the study of neurodegenerative diseases [198,199]. In some of the most common disorders, p53 activity and expression increase in human and mouse model brains [200,201,202,203]. Interestingly, in AD or PD disease, besides high p53 levels, the subcellular location is compromised in the diseased brain [204]. It was observed that whereas p53 and its phosphorylated form (p-p53) are found mainly in the nucleus of control patients, in the brain of a patient with AD, it is located almost exclusively in the cytoplasm, revealing an altered transport cytoplasm-nucleus that might be relevant for the involvement of p53 in the pathological progression of neurological disorders [204]. The formation of p53 aggregates and the destabilization of the microtubules network in the perinuclear area of neurons could be one of the reasons for this cytoplasm-nucleus transport.

Cytoplasmic p53 has been involved in both tau and amyloid pathologies in AD brains. In humans, p53 interacts with Tau and PS1. Several AD mouse models showed that Tau, APP, and PS1 expression levels modulate p53 expression. In this regard, genetic deletions of PS1 and APP decrease the expression of p53 in the mouse brain [198,205]. Increasing evidence showed that p53 is also a repressor of the activity of the different genes involved in the development of neurodegenerative diseases, such as Tau and PS1 in AD [206], and Parkin and α-synuclein in PD. p53 controls the expression of these genes and thus, in turn, can modulate the activity of p53, creating a regulatory loop where higher levels of p53 lead to a repression of PS1, Tau, or Parkin with a concomitant decrease of p53 activity. Alterations in the expression of PS1, Tau, or Parkin led to an imbalance in the regulation of p53 that could have important pathological consequences [199,204,205].

The role of p53 at the onset and development of HD has been described in depth. p53 levels are high in the brains of HD patients, and its expression also positively correlates with the grade of the disorder [207]. It has been found that p53 binds HTT. Mouse models overexpressing mutant forms of HTT.

Present increased levels of p53. In these models, genetic deletions of p53, rescued neurodegeneration showed in HD and the neurodevelopmental abnormalities associated with these models [207,208].

In PD, p53 is an important player in dopaminergic cell loss. Specific ablation of p53 in dopaminergic neurons protects against MPTP-induced neurodegeneration and improves the motor coordination found in this pharmacological approach of PD [209].

### 2.9. Cell Cycle Pathway

The cell cycle is a complex and orchestrated process that ensures duplication of the genetic material and cell division [210]. This pathway is highly regulated to avoid the transmission of the altered genome to daughter cells. There are checkpoints to regulate the cell cycle, inducing arrests for cell cycle progression and promoting DNA repair or, in case of unrepairable damage, stimulating cell death. The cell cycle consists of four phases: G0/G1, S, G2, and M. The progression is through CDKs and cyclin proteins activation by phosphorylation. In cancer cells, the aberrant activity of the cell cycle is due to mutations in genes encoding cell cycle proteins or components of upstream signaling pathways. For example, CDKN2A (encodes tumor suppressors p16^INK4A^ and p14^ARF^) and CDKN2B genes (encodes tumor suppressors p16^INK4A^-p14^ARF^ and p15^INK^, respectively) are commonly deleted, or its promoter is silenced by methylation in human cancers [210,211]. Around 54% of glioblastomas present deep deletion of cdkn2a or cdkn2b genes. CDK4 and cyclin D1 (CCDN1) locus is frequently amplified in human cancers. CDK4 is amplified in 20% of glioblastomas and sarcomas and CCDN1 is amplified in 32% of esophageal adenocarcinoma [36,171,172].

Despite neurons being postmitotic cells, and hence they are in a quiescent state, dysregulations in their cell cycle have been observed in many of the most common neurodegenerative disorders [212,213]. Neurons of diseased brains have an aberrant DNA replication. These neurons can re-enter into the cell cycle and start their DNA replication but are not able to divide. The absence of mitosis has a detrimental effect on the mature neurons, which may be associated with the development of pathologies concomitant to neurological disorder’s progress [212]. Changes in different cyclins, CDKs, and related genes have been found in postmortem brains of AD, PD, HD, and ALS patients (reviewed in [214]). In different areas of the AD brain, cyclin B, D, CDK4, and CDK5 levels, among others, are upregulated [214,215]. The activators of the CDKs are also highly expressed in AD [214,216]. Moreover, CDK2, CDK5, PCNA, and Rb are expressed aberrantly in the SN of PD patients, and HD brains present increased cyclin D1 levels [217]. The expression of mutant htt leads to neurons re-entering the cell cycle [218].

In mouse models the potential involvement was also confirmed of cell cycle re-entry found in human brains with different diseases. In AD, both pharmacologic and genetic induction of Aβ accumulation in the mouse brain triggers the expression of different genes involved in the cell cycle re-entry. A recent study shows increased expression of an S/G2/M marker in the hippocampus of AD patients and mouse models [213]. Interestingly, authors reported that neurons that undergo rapid cell cycle re-entry showed protective effects against amyloid-induced neuronal death. Dysregulation of the cell cycle in AD has been linked to hyperphosphorylation of Tau. CDK1, CDK2, or cyclin B colocalize with NFTs, and CDK5 expression induces the hyperphosphorylation of Tau. Disruption of the cell cycle has been proposed as a therapeutic target in AD. Genetic activation of the cell cycle in the mouse brain triggers progressive neurodegeneration associated with an increased amyloid load and NFT number [219]. In turn, pharmacological inhibition of specific genes related to the cell cycle alleviates the pathologies of AD mouse models [216].

Similar to AD, SN neurons of mouse models of PD present deregulated cell cycles. MPTP treatment induces the expression and activity of CDK5 in dopaminergic neurons, and its pharmacological inhibition attenuates the MPTP-induced dopamine cell loss [220]. α-synuclein, another hallmark of PD, has been documented to increase the expression of cyclin B [221,222].

### 2.10. PI3K/AKT/mTOR Pathway

The PI3K/AKT/mTOR (phosphatidylinositol 3-kinase/protein kinase-B/mechanistic target of rapamycin) signaling pathway is one of the most signally altered in cancer [223]. This pathway controls multiple cellular processes, including proliferation, survival, differentiation, metabolism, motility transcription, and protein synthesis [224]. In cancer, this pathway is hyperactivated by different genetic alterations, inducing tumorigenesis, proliferation, apoptosis, metastasis, EMT, stem-like phenotype, immune microenvironment, or drug resistance [225]. Very simplified, RTKs activate PI3K, which are heterodimers consisting of p110 catalytic and p85 regulatory subunits. P110 phosphorylate phosphatidylinositol-4,5-bisphosphate (PIP2) to generate phosphatidylinositol-3,4,5-trisphosphate (PIP3). PIP3 recruits AKT and PDK1. AKT is phosphorylated at Thr308 by PDK1 and at Ser473 by mTOR complex 2 (mTORC2), which increases its kinase activity. AKT phosphorylates TSC2 and TSC1 (tuberous sclerosis proteins 1 and 2) and dissociates the TSC1–TSC2 complex. The TSC1–TSC2 complex negatively regulates the activity of the kinase mTOR; therefore, AKT results in the activation of mTORC1 [226]. PTEN and INPP4B are negative regulators of PI3K activation [227]. The most predominant alterations in the PI3K pathway are activating events (mutations or amplification) in PIK3CA (gene encoding p110) and inactivation events (mutations or loss) in PTEN or PIK3R1. Less common alterations in AKT, TSC1, TSC2, LKB1, mTOR and other critical genes have also been found in cancer. 

Dysregulations in the different components of the PI3K pathway have been reported in the development of neurodegenerative diseases [228,229,230]. Although with controversy, signaling activation might happen in AD and PD. Significant activation (phosphorylation) of AKT was observed in neurons of postmortem brains of AD patients. With no reported changes in total levels of AKT, p-AKT, not only was found upregulated [231] but was also located near the nuclear envelope. Other studies reported decreased activation of the PI3K/AKT pathway in AD brains. There is more consensus in the pathway role in PD; AKT activity decreased in the postmortem brains of PD patients [232,233,234,235]. Diverse studies have shown the protective role of overexpressed AKT in PD mouse models [236,237,238].

PI3K/AKT signaling has a myriad of downstream components. Among others, mTOR and GSK3β are two AKT targets involved in the control of autophagy, amyloid aggregation, and Tau phosphorylation in the progression of neurodegenerative disorders, respectively. Activated AKT induces the phosphorylation of both mTOR and GSK3β. Whereas p-mTOR is the active form, phosphorylation represses the activity of GSk3β. PI3K/AKT/mTOR pathway has been linked to disrupted autophagic processes in the human AD brain. At the onset of AD, there were increased mTOR levels [239] and correlated with diminished levels of autophagy markers. In line with these findings, mTOR levels increased during the appearance of pathologies in an AD mouse model.

The PiI3K/AKT/GSK3β pathway has an important role in the hyperphosphorylation of Tau. GS3Kβ has been pinpointed as one of the most important players in Tau phosphorylation, and the consequent accumulation of NFTs reports linking AKT with GS3Kβ might also be confusing [239,240]. Increased levels of Akt found in AD brains correlated with lower activation of GSK3β. Inactivated GSK3β should diminish the levels of pTAU, but rather than this, it is described that pTAU levels are increased in the diseased brain, suggesting the implication of other kinases in the onset and progression of tautologies [239]. 

## 3. Concluding Remarks

**Figure 1:**
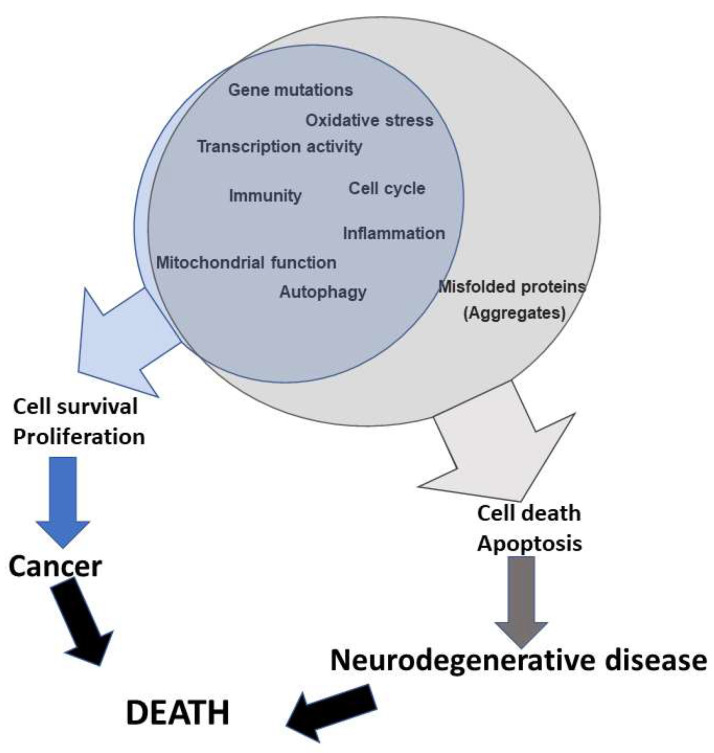
Cellular functions altered in cancer and neurodegenerative diseases. Alterations of the same molecular mechanisms can drive cell survival and proliferation in cancer and cell death and apoptosis in the development of different neurodegenerative disease.

In this review we describe the individual role and genetic alterations of the significant components of the canonical molecular cancer pathway in the context of progression of different neurodegenerative diseases (Figure 1). Effectors of these signalling pathways play crucial functions in the appearance of the most characteristic pathologies. However, the intense crosstalk between the pathways makes the identification of one individual therapeutical target complicated from a molecular point of view. Despite all the characterization of the different signalling pathways, more efforts are required to understand the global mechanisms shared by the different pathways, with the final goal of obtaining a molecular understanding of the onset and progression of neurodegenerative diseases. This would advance the development of future therapeutical treatments against sporadic degenerative diseases.

Despite this review focusing on the most altered signaling pathways in the different neurodegenerative diseases, other factors are also involved in the appearance of their pathologies. For example, the role of metabolism in the control of cognitive functions has gained relevance in the last few years [241]. It is known that alterations in energy metabolism lead to pathologies associated with neurodegeneration [241]. Peripheral and central immune systems and their crosstalk have been also pointed out as factors involved in age-related diseases [242]. Although their contribution is clear, the specific mechanisms are still not fully understood. The identification of these and other mechanisms, together with the molecular pathways, will lead to finding novel strategies for therapeutical treatments. One of the latest strategies is the implementation of stem cell therapies, whose final goal is to minimize neuronal loss [243].

## Figures and Tables

**Table 1:** Brain areas and neuronal populations affected by neurodegenerative disorders.

Neurodegenerative Disease	CNS Area	Neuronal Population	REF
Alzheimer’s disease	Hippocampus (CA1). Entorhinal Cortex. Locus coeruleus. Basal forebrain.	Pyramidal neurons. Cholinergic neurons. Noradrenergic neurons.	[14,15,16,17,18,19]
Parkinson’s disease	Substantia nigra pars compacta (SNpc). VTA (lower levels of Degeneration)	Dopaminergic neurons	[20,21,22,23]
Huntington’s Disease	Striatum	Medium spiny GABAergic neurons (MSN).	[24,25]
Amyotrophic lateral Sclerosis (ALS)	Spinal cord. Motor cortex. Brain stem.	Motor neurons	[26,27,28]
Pick’s Disease	Hippocampus Amygdala. Frontal and temporal lobes	Pyramidal and granular neurons.	[29,30,31,32]

## Data Availability

Not applicable.
